# Supplementary material for: Meta-Analysis and Gene Set Enrichment Relative to ER Status Reveal Elevated Activity of MYC and E2F in the “Basal” Breast Cancer Subgroup
Source: PLoS One. 2009 Mar 9;4(3):e4710. doi: 10.1371/journal.pone.0004710 (PMC2650420; doi:10.1371/journal.pone.0004710)
Supplement: Dataset S1 — GSEA results from first screen. The file contains a zipped archive of the HTML results of the second GSEA screen. Reports for enriched or depleted datasets may be accessed from the “index.html” link within the my_analysis.GseaPreranked.1219975950625.rpt.zip ZIP archive. Enrichment plots are available for each of the 50 most enriched and depleted gene sets. (13.32 MB ZIP) [file pone.0004710.s001.zip › my_analysis.GseaPreranked.1219975950625.rpt.zip!//DOX_RESIST_GASTRIC_UP.html]

Details for gene set DOX\_RESIST\_GASTRIC\_UP[GSEA]

|  || Dataset | zSco.ERneg.to.ERpos\_collapsed |
| Phenotype | NoPhenotypeAvailable |
| Upregulated in class | na\_pos |
| GeneSet | DOX\_RESIST\_GASTRIC\_UP |
| Enrichment Score (ES) | 0.70369124 |
| Normalized Enrichment Score (NES) | 2.4856334 |
| Nominal p-value | 0.0 |
| FDR q-value | 0.0 |
| FWER p-Value | 0.0 |
Table: GSEA Results Summary

  

Fig 1: Enrichment plot: DOX\_RESIST\_GASTRIC\_UP      
 Profile of the Running ES Score & Positions of GeneSet Members on the Rank Ordered List

  

| PROBE | GENE SYMBOL | GENE\_TITLE | RANK IN GENE LIST | RANK METRIC SCORE | RUNNING ES | CORE ENRICHMENT || 1 | CDC20 |  |  | 9 | 7.151 | 0.0439 | Yes |
| 2 | FOXM1 |  |  | 21 | 6.760 | 0.0852 | Yes |
| 3 | NCAPG |  |  | 29 | 6.628 | 0.1260 | Yes |
| 4 | BUB1 |  |  | 41 | 6.445 | 0.1654 | Yes |
| 5 | CENPA |  |  | 51 | 6.315 | 0.2041 | Yes |
| 6 | DKFZP762E1312 |  |  | 61 | 6.161 | 0.2418 | Yes |
| 7 | MKI67 |  |  | 98 | 5.871 | 0.2757 | Yes |
| 8 | MCM4 |  |  | 180 | 5.436 | 0.3035 | Yes |
| 9 | CDCA8 |  |  | 194 | 5.328 | 0.3357 | Yes |
| 10 | TPX2 |  |  | 231 | 5.180 | 0.3653 | Yes |
| 11 | KIF4A |  |  | 266 | 5.031 | 0.3941 | Yes |
| 12 | OIP5 |  |  | 320 | 4.868 | 0.4205 | Yes |
| 13 | RFC4 |  |  | 370 | 4.741 | 0.4463 | Yes |
| 14 | MAD2L1 |  |  | 477 | 4.446 | 0.4661 | Yes |
| 15 | PLK4 |  |  | 524 | 4.292 | 0.4894 | Yes |
| 16 | PRC1 |  |  | 543 | 4.253 | 0.5145 | Yes |
| 17 | DCC1 |  |  | 567 | 4.206 | 0.5390 | Yes |
| 18 | NCAPG2 |  |  | 677 | 3.978 | 0.5556 | Yes |
| 19 | TYMS |  |  | 829 | 3.738 | 0.5675 | Yes |
| 20 | ABCC1 |  |  | 877 | 3.666 | 0.5869 | Yes |
| 21 | GINS1 |  |  | 902 | 3.632 | 0.6077 | Yes |
| 22 | PLK1 |  |  | 988 | 3.499 | 0.6231 | Yes |
| 23 | CDC2 |  |  | 1259 | 3.182 | 0.6226 | Yes |
| 24 | ASF1B |  |  | 1319 | 3.119 | 0.6376 | Yes |
| 25 | CENPM |  |  | 1453 | 2.995 | 0.6463 | Yes |
| 26 | ATAD2 |  |  | 1532 | 2.917 | 0.6586 | Yes |
| 27 | HMMR |  |  | 1608 | 2.843 | 0.6707 | Yes |
| 28 | KIAA1794 |  |  | 1724 | 2.747 | 0.6791 | Yes |
| 29 | CDKN3 |  |  | 1827 | 2.674 | 0.6881 | Yes |
| 30 | PBK |  |  | 1853 | 2.647 | 0.7027 | Yes |
| 31 | RRM2 |  |  | 2047 | 2.485 | 0.7037 | Yes |
| 32 | POLA2 |  |  | 2649 | 2.117 | 0.6716 | No |
| 33 | LMNB1 |  |  | 2764 | 2.060 | 0.6759 | No |
| 34 | KIAA0286 |  |  | 3022 | 1.923 | 0.6685 | No |
| 35 | BARD1 |  |  | 3028 | 1.920 | 0.6801 | No |
| 36 | CDC6 |  |  | 3185 | 1.847 | 0.6799 | No |
| 37 | TOP2A |  |  | 3735 | 1.610 | 0.6486 | No |
| 38 | HMGB2 |  |  | 3883 | 1.558 | 0.6472 | No |
| 39 | KIAA0101 |  |  | 4770 | 1.230 | 0.5882 | No |
| 40 | RRM1 |  |  | 4872 | 1.197 | 0.5880 | No |
| 41 | NPIP |  |  | 10855 | -1.433 | 0.1464 | No |
| 42 | FOSB |  |  | 12385 | -3.084 | 0.0505 | No |
| 43 | NQO1 |  |  | 12453 | -3.182 | 0.0653 | No |
Table: GSEA details [plain text format]

  

Fig 2: DOX\_RESIST\_GASTRIC\_UP: Random ES distribution      
 Gene set null distribution of ES for **DOX\_RESIST\_GASTRIC\_UP**
